# Editorial: Novel natural therapies for infectious diseases using computational and pharmacological approaches

**DOI:** 10.3389/fphar.2026.1826975

**Published:** 2026-04-08

**Authors:** Antony Stalin, Pachaiyappan Saravana Kumar, Claudio Ferrante, Abd El-Latif Hesham, Savarimuthu Ignacimuthu, Ximei Luo

**Affiliations:** 1 Institute of Fundamental and Frontier Sciences, University of Electronic Science and Technology of China, Chengdu, China; 2 Yangtze Delta Region Institute (Quzhou), University of Electronic Science and Technology of China, Quzhou, Zhejiang, China; 3 Key Laboratory of Precise Synthesis of Functional Molecules of Zhejiang Province, Department of Chemistry, School of Science and Research Center for Industries of the Future, Westlake University, Hangzhou, China; 4 Botanic Garden “Giardino dei Semplici”, Department of Pharmacy, “G. d’Annunzio” University, Chieti, Italy; 5 Genetics Department, Faculty of Agriculture, Beni-Suef University, Beni-Suef, Egypt; 6 Xavier Research Foundation, St Xavier’s College, Palayamkottai, India

**Keywords:** artificial intelligence, bioinformatics (computational biopharmaceutics and modeling), infectious diseases, natural compounds, network pharmacology, pharmaceutical chemistry

Infectious diseases remain a persistent global public health challenge, driven by antimicrobial resistance (AMR) and the ongoing emergence of novel viral pathogens. Drug-resistant bacteria were directly responsible for approximately 1.27 million deaths in 2019, with projections indicating a significant increase in this toll without new therapeutic interventions (Murray et al., 2022). Simultaneously, the rapid mutational dynamics of RNA viruses, including SARS-CoV-2 and influenza, continually generate immune-evasive and drug-resistant variants, necessitating broad-spectrum therapeutics (Dhama et al., 2023; Zhao et al., 2023). In this context, naturally derived products from plants, fungi, and marine organisms have re-emerged as historically validated sources of novel anti-infectives (Newman and Cragg, 2020). The pharmacological potential of natural products stems from their diverse secondary metabolites that target key viral and bacterial enzymes, including proteases, polymerases, and cell wall biosynthesis (Atanasov et al., 2021). The integration of computational methodologies such as molecular docking, MD simulations, machine learning, and network pharmacology with experimental pharmacology has catalyzed a paradigm shift in natural product-based drug discovery, enabling systematic virtual screening, ADMET profiling, and mechanistic hypothesis generation (Lluka and Stokes, 2022; Stalin et al., 2024; Wang et al., 2025).

Within this context, the Research Topic “Novel Natural Therapies for Infectious Diseases Using Computational and Pharmacological Approaches” presents six articles, including four original research papers and two reviews, reflecting the breadth of contemporary natural anti-infective discovery.

Zheng et al. present a review of traditional Chinese medicine in viral pneumonia, focusing on COVID-19-related mechanisms. Instead of viewing herbal medicine as a Research Topic of remedies, the authors organize the literature by signaling pathways, including PI3K/Akt, NF-κB, JAK/STAT, and mTOR. This pathway-centered approach is valuable because it connects complex formulations to biological processes such as inflammation control, immune regulation, apoptosis, and tissue injury. The review also highlights a methodological direction for the field: natural therapies are more likely to achieve translational value when their effects are described in mechanistic terms that can be compared, tested, and refined.

A related respiratory theme is explored by Fu et al. who investigate a combination of cordycepin, lactoferrin, and *Sargassum fusiforme* polysaccharides in a murine model of RSV infection. Their study demonstrates that the combination reduces lung injury markers, lowers viral burden, and shifts macrophage responses toward an M2-like phenotype. The work is notable because it goes beyond reporting antiviral benefits and also investigates immune modulation as part of the therapeutic mechanism. This is important for RSV and other respiratory infections, where pathology is influenced not only by viral replication but also by host responses.

Vimalanathan et al. also address respiratory infection, focusing on virus-bacterial interactions in pediatric airway tissue. Using an *ex vivo* EpiAirway model, the authors show that RSV and HPIV3 promote bacterial adhesion by upregulating host receptors such as PAFr, ICAM-1, and CEACAM-1, while *Echinacea purpurea* extract reduces this dysregulation. The study provides a mechanistic explanation for how a botanical preparation may reduce respiratory complications and downstream antibiotic use. This is a valuable contribution because it links natural-product intervention to a clinically relevant problem at the intersection of viral infection, bacterial superinfection, and antimicrobial stewardship.

Beyond respiratory disease, Ghasemian Yadegari et al. investigate *Astragalus onobrychis L*. extract against *Echinococcus granulosus*. Their study integrates *in vitro*, *ex vivo*, and *in vivo* analyses, demonstrating scolicidal activity with evidence of apoptosis induction, DNA damage, and reduced cyst burden in infected mice. By linking phenotypic efficacy with caspase-3 activation and upregulation of EgATM and EgP53, the authors advance descriptive antiparasitic screening toward mechanistic pharmacology. This approach is valuable in parasitology, where many plant extracts are reported to be active, but fewer studies clarify the underlying mechanisms of action.

Javid et al. present an antimicrobial study of *Bistorta amplexicaulis* D. that combines phytochemical profiling, *in vitro* antibacterial and antifungal assays, and computational analysis. Their data show that plant extracts, especially ethyl acetate fractions, contain bioactive constituents and display measurable activity against bacterial and fungal pathogens; while docking and molecular dynamics simulations support plausible interactions with microbial targets. The strength of this work lies in its integrated workflow: metabolite characterization, biological testing, and *in silico* interpretation are used together rather than as isolated steps. This design increases the value of exploratory phytochemical studies by making the biological observations more interpretable.

At the discovery-platform level, Narsing Rao et al. review omics strategies for obtaining molecules from marine *Actinomycetota*. The article highlights genome mining, metagenomics, transcriptomics, metabolomics, heterologous expression, and analytical methods as tools for overcoming a problem in natural product research: the repeated rediscovery of known compounds. Their review is relevant to this Research Topic because it expands the discussion from individual herbal interventions to the infrastructure needed for natural product discovery. In doing so, it reinforces a central message of the Research Topic: progress in infectious disease pharmacology will increasingly depend on how well biological resources, computational methods, and validation strategies are integrated.

The six articles demonstrate that the discovery of natural therapies for infectious diseases is shifting from single-technique, single-endpoint studies toward more integrated workflows. Several priorities emerge from this Research Topic. First, natural products research in infectious disease requires stronger mechanistic resolution, so that extracts or compounds can be linked to defined pathways, host responses, or microbial targets. Second, computational tools should serve as decision-support systems that improve prioritization and reduce experimental ambiguity, rather than functioning as stand-alone additions. Third, model systems are important: clinically relevant *ex vivo* models, immune-aware infection models, and omics-guided microbial discovery platforms can enhance translational value. Finally, the field would benefit from improved standardization of phytochemical characterization, endpoint reporting, toxicity assessment, and data sharing, which are necessary for reproducibility and meaningful comparison across studies.

Overall, this Research Topic provides a perspective on how natural therapies, when studied with pharmacological rigor and computational support, can contribute to anti-infective discovery. The Research Topic does not claim that natural products will resolve the current burden of infectious disease. Instead, it presents a clearer argument: natural compounds, herbal formulations, and microbially derived metabolites remain therapeutic resources, and their future value will be greatest when discovery is guided by mechanism, informed by data, and validated in relevant systems.
